# Age-Related Changes in the Neural Processes of Reward-Directed Action and Inhibition of Action

**DOI:** 10.3389/fpsyg.2020.01121

**Published:** 2020-06-10

**Authors:** Thang M. Le, Herta Chao, Ifat Levy, Chiang-Shan R. Li

**Affiliations:** ^1^Department of Psychiatry, Yale University School of Medicine, New Haven, CT, United States; ^2^Department of Medicine, Yale University School of Medicine, New Haven, CT, United States; ^3^VA Connecticut Healthcare System, West Haven, CT, United States; ^4^Department of Comparative Medicine, Yale University School of Medicine, New Haven, CT, United States; ^5^Department of Neuroscience, Yale University School of Medicine, New Haven, CT, United States; ^6^Interdepartmental Neuroscience Program, Yale University School of Medicine, New Haven, CT, United States

**Keywords:** aging, action, inhibition of action, reward, fMRI

## Abstract

Aging is associated with structural and functional brain changes which may impact the regulation of motivated behaviors, including both action and inhibition of action. As behavioral regulation is often exercised in response to reward, it remains unclear how aging may influence reward-directed action and inhibition of action differently. Here we addressed this issue with the functional magnetic resonance imaging data of 72 participants (aged 21–74) performing a reward go/no-go (GNG) task with approximately 2/3 go and 1/3 no-go trials. The go and no-go success trials were rewarded with a dollar or a nickel, and the incorrect responses were penalized. An additional block of the GNG task without reward/punishment served as the control to account for age-related slowing in processing speed. The results showed a prolonged response time (RT) in rewarded (vs. control) go trials with increasing age. Whole-brain multiple regressions of rewarded (vs. control) go trials against age and RT both revealed an age-related reduced activity of the anterior insula, middle frontal gyrus, and rostral anterior cingulate cortex. Furthermore, activity from these regions mediated the relationship between age and go performance. During rewarded (vs. control) no-go trials, age was associated with increased accuracy rate but decreased activation in the medial superior frontal and postcentral gyri. As these regions also exhibited age-related activity reduction during rewarded go, the finding suggests aging effects on common brain substrates that regulate both action and action inhibition. Taken together, age shows a broad negative modulation on neural activations but differential effects on performance during rewarded action and inhibition of action.

## Introduction

Aging is associated with changes in brain structures and functions including those underlying goal-directed behaviors (Marschner et al., 2005). Previous imaging evidence suggests a negative relationship between age and neural activities during tasks involving action and inhibition of action. For instance, older individuals showed a diminished primary motor cortical activation to forceful hand grips (Ward et al., 2008) and sequential movements (Sharma and Baron, 2014). In inhibition of action, age was associated with an attenuated activity in the left orbitofrontal and dorsolateral prefrontal cortex during successful vs. unsuccessful stopping in the stop-signal task (Hu et al., 2012). Voxel-based morphometry further demonstrated gray matter volume reductions in the right dorsolateral prefrontal cortex, caudate head, and bilateral insula with age (Hu et al., 2018). These age-related neural alterations likely have behavioral implications. Indeed the latter study reported a relationship between age-related changes in the regional gray matter volume in these regions and prolonged stop-signal reaction time, an index of behavioral inhibition. Other studies showed that older, as compared to young, adults were slower in initiating actions in a two-choice decision (Eppinger et al., 2012), Stroop (Verhaeghen and De Meersman, 1998; Zysset et al., 2007), and stop-signal (Rush et al., 2006; Hu et al., 2019) tasks. Past evidence together suggests decreasing brain activities and weaker task performance during action and inhibition of action with advancing age.

Goal-directed behaviors are frequently driven by reward. As aging may alter reward responses, it is critical to understand how age influences reward processing during action regulation. Aging appears to broadly diminish both behavioral and brain responses to reward. During the reinforcement learning task, a study using an electroencephalogram reported that feedback-related negativity, an event-related potential in response to negative feedback, for monetary gains showed a monotonic reduction from childhood to old age (Hämmerer et al., 2011), suggesting age-related diminution in reward-related saliency response. In the monetary incentive delay task, age was negatively correlated with neural activations to anticipated large vs. small rewards in regions implicated in reward processing, including the orbitofrontal cortex and ventral striatum (Dhingra et al., 2019). These findings support previous reports of reduced dopaminergic signaling and reward sensitivity in aging (Kish et al., 1995; Volkow et al., 1996; Kumakura et al., 2010). In behavioral investigations, older adults exhibited decreased sensitivity to reward (SR) probability in a signal-detection task (Tripp and Alsop, 1999), greater risk aversion in economic decision tasks (Grubb et al., 2016; Mata et al., 2016; Rutledge et al., 2016), and less delay discounting (Green et al., 1994) as compared to young adults. Although older adults showed reduced sensitivity to winning a large vs. small reward, they were more sensitive to the loss of a small vs. large reward as compared to younger adults (Dhingra et al., 2019). These age-related changes in reward and risk/loss sensitivity likely influence motivated action and inhibition of action, respectively. One possibility is that diminished SR negatively impacts reward-directed action, manifesting in age-related decreases in brain responses. In contrast, higher sensitivity to loss or risk may be associated with increases in activations to inhibition of action in older relative to younger adults.

As motor slowing is commonly observed in aging (Salthouse, 2000), it is important to consider age-related changes in processing speed when examining changes in goal-directed behaviors. Previous work has associated age with motor slowing in the stop-signal task (Rush et al., 2006) as well as in both congruent and incongruent conditions in a Stroop task (Verhaeghen and De Meersman, 1998), indicating a general slowing in behavioral responses. Age-related decreases in processing speed also negatively affected response time (RT) in tasks that engaged memory, verbal, and spatial processing during cognitive control (Finkel et al., 2007). As such, general motor slowing may account for some of the changes in goal-directed behaviors in older adults. Nevertheless, no study, to our knowledge, has controlled for this motor component when examining how age may alter the neural substrates that support rewarded action and inhibition of action.

To investigate how age influences motivated action and inhibition of action, we employed a go/no-go (GNG) task in which both correct action and inhibition of action were rewarded and both incorrect trials were penalized. As reward/punishment sensitivity changes with age, we used two different monetary values, dollar and nickel, to explore potential differences related to win/loss magnitude in behavioral and neural responses. We included a no reward/punishment condition as a baseline to control for age-related decline in processing speed. Individual differences in reward and punishment sensitivity were further controlled to better identify age-specific effects. We tested the hypothesis that rewarded go performance as well as neural activations to go responses would diminish with age and that rewarded no-go performance and neural activations to no-go responses would both be enhanced with age. Finally, we used mediation models to investigate the inter-relationships between age, behavioral performance, and brain activations while controlling for processing speed and trait sensitivities.

## Materials and Methods

### Participants and Assessments

Seventy-two healthy adults (36 females; age range = 21–74; mean ± SD, 36.4 ± 13.9 years) participated in the study. All the subjects were screened to ensure absence of major medical, including neurological, illness and lifetime Axis I psychiatric disorders. No participant was currently on psychotropic medications and all tested negative for illicit substances on the study day. The subjects provided written informed consent after the details of the study were explained, in accordance to institute guidelines approved by the Yale Human Investigation Committee.

All the participants completed the Sensitivity to Punishment and Sensitivity to Reward Questionnaire (Torrubia et al., 2001), which contains 48 yes–no items, with 24 items measuring behavioral impulsivity/responsiveness to reward and the other 24 measuring behavioral avoidance in response to potentially adverse consequences. The scores were obtained by totaling the number of yes answers in each scale, with higher subscores indicating greater SR and sensitivity to punishment (SP), respectively. The participants reported averages in SR score of 9.79 ± 4.73 and SP score of 8.58 ± 5.33.

### Behavioral Task

**FIGURE 1:**
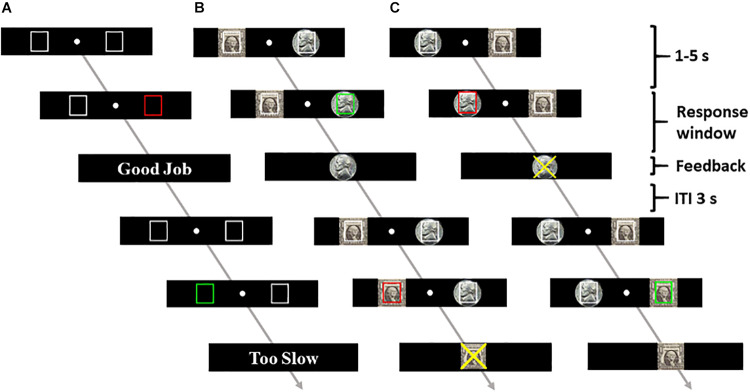
Task schematic. The participants performed a go/no-go task for one control session (session 3) with no reward **(A)** and four sessions with the dollar reward on the left **(B)** and on the right **(C)**, counter-balanced in order across subjects. A successful go trial and a failed no-go trial are illustrated in both **(B)** and **(C)**.

The participants performed a GNG task, completing two reward runs, followed by one control and two additional reward runs (Figure 1). In the reward runs, a dollar image and a nickel image were presented to the left/right of the fixation in two runs and were reversed in direction for the other two, with the order counter-balanced across subjects. Go (∼66.6%) and no-go (∼33.3%) trials were randomly intermixed in presentation, with an inter-trial interval of 3 s.

At the beginning of each trial, two black squares (control run) or two images, one of dollar and one of nickel (reward runs), appeared to the left and right of the fixation. The squares and the images were outlined in white. After a randomized interval between 1 and 5 s, one of the outlines (i.e., either left or right square/image) turned green/red, indicating a GNG signal. The subjects were instructed to press the spatially corresponding left/right button as quickly as possible in response to the go signal and to withhold the button press to the no-go signal. Feedback was provided at button press or once the response window had elapsed. In the control run, the feedback displayed the text “Good Job” for correct responses or “Too Slow” and “Don’t” for incorrect go and no-go responses, respectively. For the reward runs, the participants won a dollar or a nickel in each correct trial. An image of a dollar or nickel was shown as feedback to indicate the amount. In incorrect trials, the participants lost either a dollar or a nickel. A symbol “X” overlaid on the dollar or the nickel image informed of the amount of money loss. Premature button presses prior to the color change were treated as errors, resulting in the feedback “Don’t” for control trials or symbol X overlaid on the dollar/nickel for reward trials. The participants performed approximately 50 go and 25 no-go trials in the control run and 100 go and 50 no-go each of the dollar-and-nickel trials in the reward runs. The participants won an average of $123 ± 38 (mean ± SD).

Prior to imaging, the subjects completed a control session outside the scanner. A normal distribution function was fitted on the RT, and 10^7^ data points were generated based on the fitted function. The response window for go success was set as the closest integer greater than 85% of the generated data points for the fMRI experiment.

### Behavioral Analysis

The trial types were separated for the go and the no-go responses as well as the dollar and the nickel rewards: GS dollar, GS nickel, GE dollar, GE nickel, NGS dollar, NGS nickel, NGE dollar, and NGE nickel (GS: go success; GE: go error; NGS: no-go success; and NGE: no-go error). The number of trials for each condition and each block is detailed in Supplementary Table S1. For the response rate, a two-way (GS vs. NGS × dollar vs. nickel vs. control) analysis of variance (ANOVA) was conducted. *Post hoc* comparisons were performed to further investigate the potentially differential effects of reward on action and inhibition of action. Similarly, we used a two-way (GS vs. NGE × dollar vs. nickel vs. control) ANOVA to examine the RT. Subjects without NGE trials were omitted from the analysis. To examine the relationship between age and task performance, we performed partial correlations using sex, SR, and SP as covariates. We controlled for task performance in the control session to account for general age-related changes in processing speed. For instance, the RT of the go control trials was subtracted from the go dollar trials (e.g., GS dollar RT - GS control RT) in the analyses involving go dollar RT.

### Imaging Protocol and Data Preprocessing

Conventional T1-weighted spin echo sagittal anatomical images were acquired for slice localization using a 3T scanner (Siemens Trio, Erlangen, Germany). Anatomical 3D MPRAGE images were obtained with spin echo imaging in the axial plane parallel to the anterior commissure–posterior commissure (AC–PC) line with repetition time (TR) = 1,900 ms, echo time (TE) = 2.52 ms, bandwidth = 170 Hz/pixel, field of view (FOV) = 250 mm × 250 mm, matrix = 256 × 256, 176 slices with slice thickness = 1 mm, and no gap. Functional blood oxygenation level-dependent (BOLD) signals were acquired using multiband imaging (multiband acceleration factor = 3) with a single-shot gradient echo-planar imaging sequence. Fifty-one axial slices, parallel to the AC–PC line covering the whole brain, were acquired with TR = 1,000 ms, TE = 30 ms, bandwidth = 2290 Hz/pixel, flip angle = 62°, FOV = 210 mm × 210 mm, matrix = 84 × 84, voxel size = 2.5 mm isotropic, and no gap.

The imaging data were preprocessed using SPM12 (Wellcome Trust Centre for Neuroimaging). Images from the first five TRs at the beginning of each run were discarded to ensure that only BOLD signals at steady-state equilibrium between radio frequency pulsing and relaxation were included in the analyses. The images of individual subjects were first realigned (motion-corrected) and corrected for slice timing. A mean functional image volume was constructed for each subject per run from the realigned image volumes. These mean images were co-registered with the high-resolution structural image and then segmented for normalization with affine registration followed by nonlinear transformation. The normalization parameters determined for the structure volume were then applied to the corresponding functional image volumes. The voxel size after normalization was 2.5 mm isotropic. Finally, the images were smoothed with a Gaussian kernel of 4-mm full width at half-maximum.

### Imaging Data Modeling

A statistical analytical design was constructed for individual subjects using the general linear model (GLM), with the onsets of go or no-go signals convolved with a canonical hemodynamic response function (HRF) and with the temporal derivative of the canonical HRF and entered as regressors in the model (Friston et al., 1995). As go and no-go error trials were associated with an RT, a column of RT was entered as a parametric modulator each for GS, GE, and NGE trials in the model. Realignment parameters in all six dimensions were also entered in the model. The data were high-pass-filtered (128-s cutoff) to remove low-frequency signal drifts. Serial autocorrelation caused by aliased cardiovascular and respiratory effects was corrected by a FAST model. The GLM estimated the component of variance that could be explained by each of the regressors.

In the first-level analysis, we constructed for the statistical contrasts required for second-level analyses. To examine how brain activities associated with action and inhibition of action varied across subjects in relation to age and behavioral performance, we conducted whole-brain multiple regressions against age with sex and the SR and SP scores as the covariates. Specifically, we examined the contrasts (GS dollar > GS control) and (NGS dollar > NGS control) in correlation with age. We used the GS/NGS control as the baseline to account for age-related changes in processing speed which could confound any potential alterations in approach and avoidance. Similarly, sex and the SR and SP scores were used as covariates to rule out the effects of individual differences in gender and trait sensitivities. We further investigated the neural correlates of go performance by conducting a whole-brain multiple regression for the (GS dollar > GS control) contrast against (GS dollar RT - GS control RT), again using sex and trait sensitivities as the covariates. For no-go trials, as no RT was available, we used (NGS dollar accuracy rate - NGS control accuracy rate) and (NGS dollar > NGS control) contrast. Another set of analysis was conducted for the nickel condition. Cohen *f*^2^ values were calculated to measure the effect size of multiple linear regressions with small (0.02), medium (0.15), and large (0.35) effects consistent with interpretation guidelines (Cohen, 1992). All regression results were examined with voxel *p* < 0.001 in combination with cluster *p* < 0.05, corrected for family-wise error, according to current reporting standards (Woo et al., 2014; Eklund et al., 2016). All activations were reported in Montreal Neurological Institute coordinates.

### Mediation Analysis

To examine the inter-relationships of age, neural activity, and task performance, we conducted mediation analyses using a single-mediator model (MacKinnon et al., 2007). The methods were detailed in our previous work (Le et al., 2019a, b). Briefly, in a mediation analysis, the relationship between the independent variable *X* and dependent variable *Y* (i.e., *X* → *Y*) is tested to determine whether it is significantly mediated by a variable *M*. The mediation test is performed using the following three regression equations:

Y=i⁢1+c⁢X+e⁢1

$$Y=i\,2+c^{\prime }\,X+b\,M+e\,2$$

M=i⁢3+a⁢X+e⁢3

where a represents *X* → *M*, b represents *M* → *Y* (controlling for *X*), *c*’ represents *X* → *Y* (controlling for *M*), and c represents *X* → *Y*. *a*, *b*, *c*, and *c*’ are path coefficients. Variable *M* is said to serve as a mediator of connection *X* → *Y* if (*c* – *c*’) is significantly different from zero (MacKinnon et al., 2007). If (*c* – *c*’) is different from zero and the paths *a* and *b* are significant, then *X* → *Y* is mediated by *M*. Additionally, if path *c*’ is not significant, there is no direct connection from *X* to *Y*, in which case *X* → *Y* is completely mediated by *M*. The analysis was performed with package Lavaan (Rosseel, 2012) in R. To test the significance of the mediation effect, we used the bootstrapping method (Preacher and Hayes, 2004) as it is generally considered advantageous to the Sobel test (MacKinnon et al., 2007).

Specifically, we evaluated the inter-relationships between age, task performance, and neural activity of GS dollar > GS control (see section “Results”). For neural activity, we extracted the parameter estimates (effect size) from the overlapping voxels of the two multiple regressions of (GS dollar > GS control) against age and (GS dollar RT > GS control RT). We considered three models (Figure 4). In model 1, age served as the independent variable (*X*), RT as the dependent variable (*Y*), and neural activity as the mediator (*M*). Thus, age contributed to neural activity, which in turn modulated task performance: age → neural activity → RT. In model 2, age contributed to neural activity and this relationship was mediated by task performance: age → RT→ neural activity. In model 3, neural activity contributed to task performance and this relationship was mediated by age: neural activity → age → RT. We did not consider the remaining three models in which age or neural activity served as the dependent variable as these models lacked conceptual import. We used Bonferroni (*p* = 0.017) to correct for multiple-model testing.

## Results

### The Effects of Age on Behavioral Performance

**FIGURE 2:**
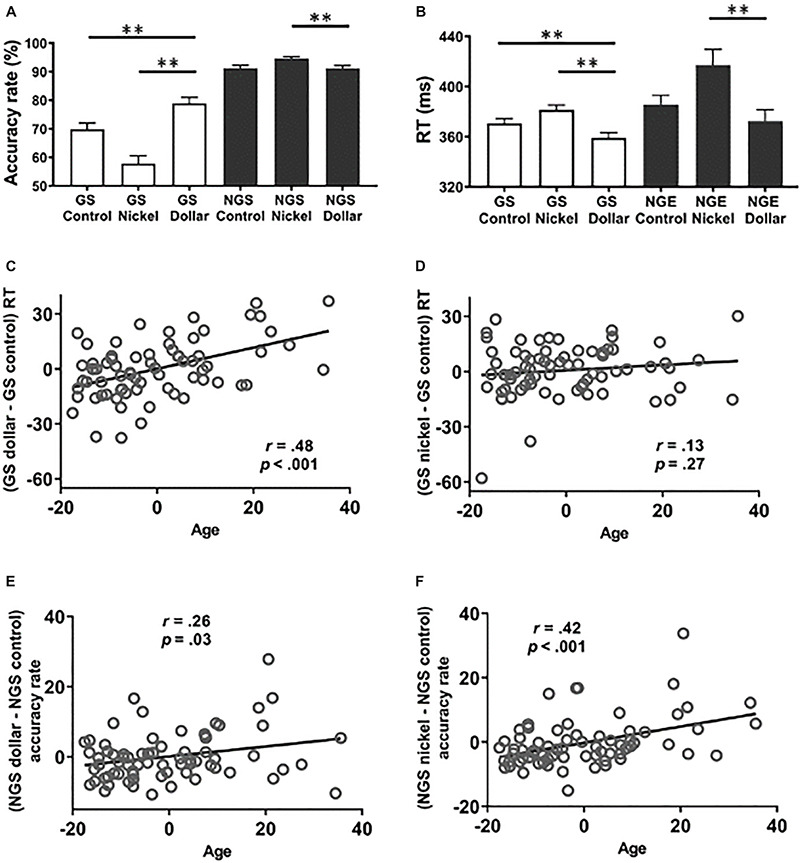
Behavioral results. Behavioral results (mean ± SE) showed the accuracy rate **(A)** and the reaction time **(B)** across trial types. Age was positively correlated with response time of the go responses in the dollar **(C)** but not nickel **(D)** vs. the control trials. Age was also positively correlated with the accuracy rate of no-go dollar **(E)** and nickel **(F)** vs. the control trials. GS, go success; NGS, no-go success; NGE, no-go error. **p ≤ 0.001. NB: all scatterplots show partial correlations of residuals after the effects of trait sensitivities and sex were removed.

Figures 2A,B show the accuracy rate and the RT across conditions. For the accuracy rate, a two-way (GS vs. NGS × dollar vs. nickel vs. control) ANOVA showed a significant main effect of response [*F*(1, 426) = 246.01, *p* < 0.001, partial η^2^ = 0.37], reward value [*F*(2, 426) = 12.10, *p* < 0.001, partial η^2^ = 0.05], and response × reward value interaction [*F*(2,426) = 23.71, *p* < 0.001, partial η^2^ = 0.10]. In *post hoc* analyses, the accuracy rate was significantly higher in the GS dollar than in the GS nickel and the GS control trials (*p*’s < 0.001) and higher in the GS control than in the GS nickel trials (*p* < 0.001). In contrast, the accuracy rate for NGS dollar trials was significantly lower than for the NGS nickel trials (*p* < 0.001). The NGS control rate was significantly lower than the NGS nickel rate (*p* < 0.001) but did not significantly differ from the NGS dollar rate (*p* = 0.93).

For RT, 28 subjects did not commit any error in one of the trial conditions and thus were not included in the analysis. A two-way (GS vs. NGE × dollar vs. nickel vs. control) ANOVA showed a significant main effect of response [*F*(1, 258) = 8.52, *p* = 0.004, partial η^2^ = 0.03] and reward value [*F*(2, 258) = 8.31, *p* < 0.001, partial η^2^ = 0.06] but not the response × reward value interaction [*F*(1, 246) = 1.13, *p* = 0.32, partial η^2^ = 0.008]. RT was significantly faster in the GS dollar compared to the GS nickel and the GS control trials (*p*’s < 0.001) (Figure 2B). Response time for the GS nickel trials was slower than for the GS control trials (*p* < 0.001). The RT for NGE dollar trials was significantly faster than that for NGE nickel trials (*p* < 0.001) but did not differ significantly from the NGE control trials (*p* = 0.28), and the latter two were not significantly different after controlling for multiple corrections (uncorrected *p* = 0.05).

Next, we examined the relationship between age and behavioral measures of action and inhibition of action after accounting for processing speed, trait sensitivities, and sex. The results were evaluated at a corrected *p* value of 0.05/8 = 0.006. Age was significantly and positively correlated with (GS dollar RT - GS control RT), controlling for sex and the SR and SP scores (*r* = 0.48, *p* < 0.001, Figure 2C), but not with (GS nickel RT - GS control RT) (*p* = 0.27, Figure 2D). Age did not show significant correlations with the accuracy rate of (GS dollar - GS control) or (GS nickel - GS control) (*p*’s > 0.62). There was a significant correlation between age and the accuracy rate of (NGS dollar - NGS control) (*r* = 0.26, uncorrected *p* = 0.03, not significant after correction for multiple comparisons, Figure 2E) as well as (NGS nickel - NGS control) (*r* = 0.42, *p* < 0.001, Figure 2F). Taken together, we found partial evidence for age-related impediment of action and enhancement of inhibition of action, with the former primarily in the dollar condition and the latter in the nickel condition.

### The Effects of Age on Regional Responses to Reward-Directed Action

**FIGURE 3:**
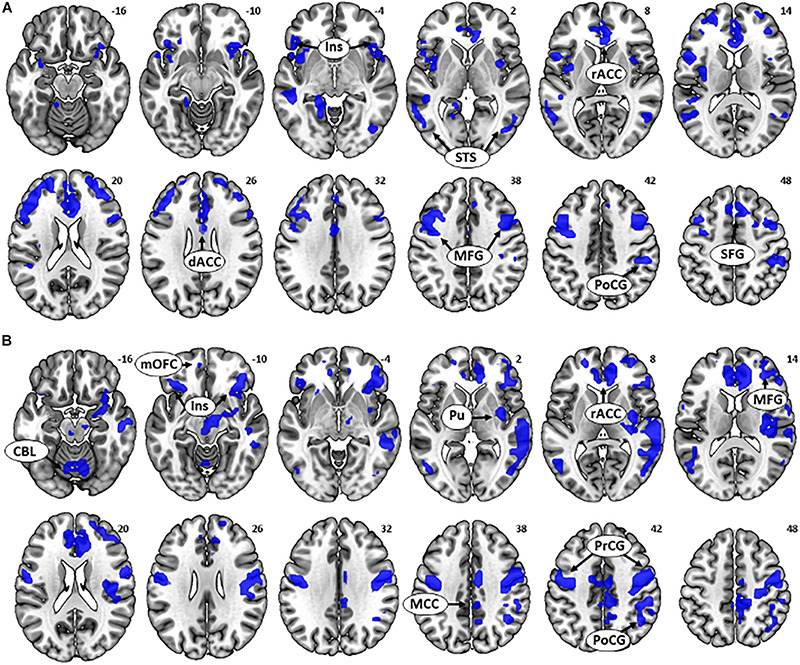
**(A)** Multiple regression for the contrast [go success (GS) dollar > GS control] showed a negative correlation between age and activations in the bilateral anterior insula, middle frontal gyri (MFG), postcentral gyrus, superior temporal sulci, rostral anterior cingulate cortex (rACC), and dorsal anterior cingulate cortex. **(B)** Multiple regression for contrast (GS dollar > GS control) showed a negative correlation between [GS dollar response time (RT) - GS control RT] and activations in the bilateral insula, medial orbitofrontal cortex, right MFG, rACC, mid-cingulate cortex, precentral gyrus/postcentral gyrus, a cluster containing the right posterior insula and putamen, and cerebellum.

As there was a significant relationship between age and (GS dollar RT - GS control RT), we focused on the GS dollar > GS control contrast. The whole-brain multiple regression against age showed a significant negative correlation with activations in the bilateral anterior insula, bilateral middle frontal gyri (MFG), superior frontal gyrus (SFG), postcentral gyrus (PoCG), bilateral superior temporal sulci, and dorsal/rostral anterior cingulate cortex (dACC/rACC) (Figure 3A and Table 1). No clusters showed an activity in significant positive correlation with age. The multiple regression for GS nickel trial showed a similar, albeit weaker, pattern of activation (Supplementary Figure S1).

**FIGURE 4:**
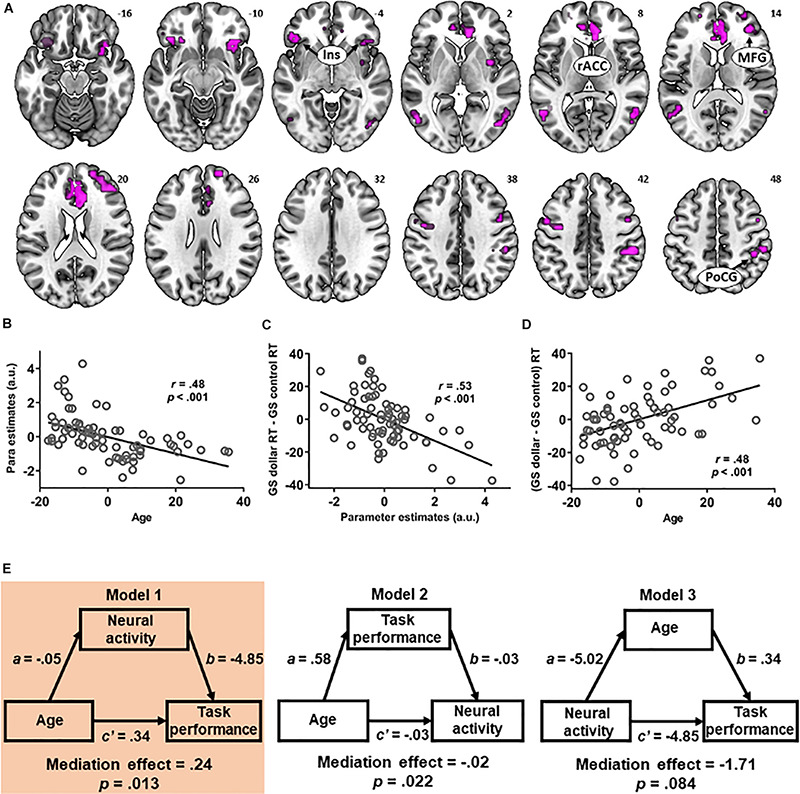
**(A)** The multiple regressions of [go success (GS) dollar > GS control] contrast against age and against [GS dollar response time (RT) - GS control RT] showed an overlap in the bilateral insula, right middle frontal gyrus, rostral anterior cingulate cortex, and dorsal anterior cingulate cortex. The activity in the overlapping voxels during (GS dollar > GS control) showed a negative correlation with **(B)** age and **(C)** (GS dollar RT - GS control RT). **(D)** The latter two showed a positive relationship, as also shown in Figure 2C. **(E)** Mediation analysis revealed a significant mediation effect in model 1 in which age was associated with prolonged RT (GS dollar RT - GS control RT), and this relationship was mediated by a diminished activity during rewarded (vs. control) action.

**TABLE 1:** Age modulation of activation to action and inhibition of action.

	Region	Montreal Neurological Institute coordinates (mm)			Voxel	Cluster
		*x*	*y*	*Z*	*T*	*k*
Go success (GS) dollar > GS control	SFG/dACC	9	17	55	5.52	2,276
	rACC	6	23	22	5.28	
	Insula	−33	−1	10	3.65	
		36	23	−14	4.52	137
		33	14	−14	3.79	
	MFG	27	59	22	4.60	168
		39	41	22	4.03	
	STS	51	−70	4	4.00	98
		−57	−58	10	3.96	127
		−48	−31	−8	4.33	119
		−48	−37	−2	4.31	
	PHG	−18	−55	−2	4.03	170
	PoCG	48	−34	46	4.20	100
		57	−37	49	3.87	
No−go success (NGS) dollar > NGS control	SFG	12	20	61	4.26	111
		12	11	67	3.91	
		6	26	55	3.75	
	PoCG	48	−43	46	3.98	106
		57	−49	46	3.70	

*dACC, dorsal anterior cingulate cortex; MFG, middle frontal gyrus; PHG, parahippocampal gyrus; PoCG, postcentral gyrus; rACC, rostral anterior cingulate cortex; SFG, superior frontal gyrus; STS, superior temporal sulcus.*

### Regional Responses to Reward-Directed Action in Correlation With RT Performance

Next, we examined the neural correlates of task performance during rewarded action. There was a negative correlation between (GS dollar RT - GS control RT) and activations to the contrast (GS dollar > GS control) in the bilateral anterior insula, mOFC, right MFG, rACC, dACC, mid-cingulate cortex, cerebellum, pre/PoCG, and a cluster containing the right posterior insula and putamen (Figure 3B and Table 2). No clusters showed an activity in positive correlation with RT.

**TABLE 2:** Neural correlates of response time during rewarded action.

Region	Montreal Neurological Institute coordinates (mm)			Voxel	Cluster
	*x*	*Y*	*z*	*T*	*k*
PrCG	51	−4	43	5.38	2,022
	−15	−28	55	5.63	
	−39	−13	40	5.25	277
	−54	−7	25	4.82	
	−54	−4	43	4.68	
rACC	12	32	19	5.16	1,258
	12	44	10	4.93	
Cerebellum	−9	−82	−35	4.96	533
	−15	−70	−38	4.84	
	−15	−61	−38	4.65	
PoCG	42	−37	40	4.59	205
	45	−55	40	4.55	
	54	−34	43	4.13	
Insula	−36	29	−11	4.49	102
	−45	29	−2	4.44	
	−27	26	−14	4.31	
STS	−54	−67	10	4.26	110
	−51	−70	1	4.19	

*dACC, dorsal anterior cingulate cortex; PoCG, postcentral gyrus; PrCG, precentral gyrus; rACC, rostral anterior cingulate cortex; STS, superior temporal sulcus.*

### Age- and Performance-Shared Correlates During Reward-Directed Action

The multiple regressions of (GS dollar > GS control) against age and against (GS dollar RT - GS control RT) revealed that voxels overlapped in the bilateral anterior insula, right MFG, rACC, and dACC (Figure 4A). Thus, we examined the inter-relationships between the activity of these overlapping voxels, age, and task performance during rewarded action. The averaged parameter estimates across these voxels were extracted for contrast (GS dollar > GS control), and, as expected, were significantly correlated with age (*r* = 0.48, *p* < 0.001, effect size = 0.30, Figure 4B) and (GS dollar RT - GS control RT) (*r* = 0.53, *p* < 0.001, effect size = 0.39, Figure 4C). As shown earlier, age and (GS dollar RT - GS control RT) showed a positive relationship (*r* = 0.48, *p* < 0.001, Figure 4D).

We conducted a mediation analysis (Table 3). Model 1 (age → neural activity → task performance) showed a significant mediation effect [*c* − *c’* = 0.24, *p* = 0.013, 95% confidence interval = (0.08, 0.45); highlighted, left]. Specifically, the path coefficient *c* (i.e., age → task performance before accounting for the mediating effect of neural activity) was significant (*p* < 0.001) and the path coefficient *c’* (i.e., after accounting for the mediating effect) was substantially weakened (*p* = 0.05). Thus, older age led to slower RT during rewarded action and the neural activity mediated this relationship. Model 2 (age → task performance → neural activity) also showed a significant mediation effect but did not survive correction for multiple-model testing (uncorrected *p* = 0.022). No significant mediation effect was found for model 3 (neural activity → age → task performance) (uncorrected *p* = 0.084).

**TABLE 3:** Mediation of age, [go success (GS) dollar response time (RT) - GS control RT], and activity during rewarded action.

	Path *a* (*X* → *M*)	Path b (*M* → *Y*)	Path *c* (*X* → *Y*)	Path *c*’ (*X* → *Y*)	Mediation path (*c* − *c*’)
Model 1: *X* (age) → *Y* (RT) mediated by *M* (neural activity)					
β	–0.05	–4.85	0.58	0.34	0.24
*p*-values	0.000	0.002	0.000	0.05	0.013
Model 2: *X* (age) → *Y* (neural activity) mediated by *M* (RT)					
β	0.58	–0.03	–0.05	–0.03	–0.02
*p*-values	0.000	0.003	0.000	0.001	0.022
Model 3: *X* (neural activity) → *Y* (RT) mediated by *M* (age)					
β	–5.02	0.34	–6.56	–4.85	–1.71
*p*-values	0.000	0.048	0.000	0.002	0.084

### The Effects of Age on Regional Responses to Reward-Directed Inhibition of Action

**FIGURE 5:**
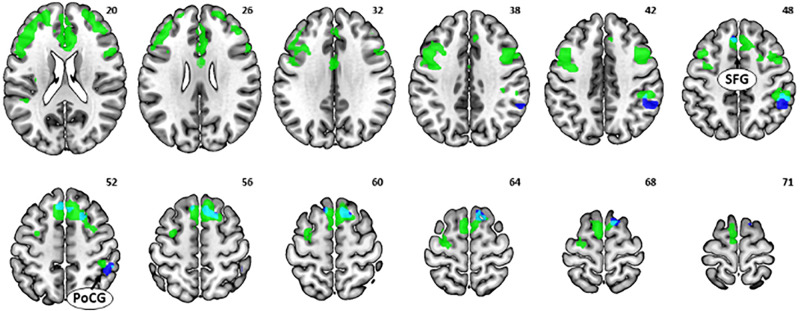
Multiple regression of [no-go success (NGS) dollar > NGS control] against age showed a negative correlation with activations in the superior frontal gyrus and postcentral gyrus (dark blue). These neural correlates showed overlap (light blue) with the multiple regression of [go success (GS) dollar > GS control] against age (green).

The multiple regression of (NGS dollar > NGS control) against age showed a significant negative correlation with activations in the bilateral SFG and right PoCG (Figure 5, dark blue and Table 1). No clusters showed a significant positive correlation with age.

It is notable that the SFG, particularly the media part, and the right PoCG showed an age-related decrease in activations to both (GS dollar > GS control) (i.e., Figure 2; shown as green in Figure 5) and (NGS dollar > NGS control) (Figure 5, light blue). This indicates aging effects on common brain substrates that may regulate both rewarded action and inhibition of action.

No clusters showed a significant correlation in the multiple regression of (NGS dollar > NGS control) against the accuracy rate of (NGS dollar - NGS control) in either direction.

The multiple regression of (NGS nickel > NGS control) against age or against the accuracy rate of (NGS nickel - NGS control) did not show clusters in significant correlation in either direction.

## Discussion

Employing a GNG task, we examined the effects of age on behavioral performance and neural processes during rewarded action and inhibition of action, controlling for individual differences in gender and reward and punishment sensitivities. Age was associated with prolonged RT and a reduction of activity in the anterior insula, MFG, rACC, and dACC during rewarded action. These regions also showed activation in negative correlation with RT, indicating a potential inter-relationship between age, neural activity, and task performance. The mediation models confirmed that increasing age diminished brain activations to goal-directed action, which in turn slowed down behavioral responses. In contrast, age was positively correlated with the accuracy rate during rewarded inhibition of action, suggesting opposing effects of age on initiating and restraining an action. There was a negative relationship between age and activations to inhibition of action in the SFG and the PoCG. Both regions also showed age-related decreases in activations during rewarded action, thus pointing to a common age correlate for the execution and the inhibition of an action.

### Age Effects on Behavioral Performance of Rewarded Action and Inhibition of Action

Age was associated with a prolonged RT of the rewarded go response, revealing a negative impact of age on motivated action, in agreement with previous investigations of the GNG task (Sebastian et al., 2013; Votruba and Langenecker, 2013). We controlled for processing speed with a neutral session to account for age-related motor slowing. Unlike most previous work using similar tasks, we incentivized responses to examine the relationship of age and reward-directed behavior. It is plausible that older adults experience a decline in motivation to act in pursuit of reward. Consistent with this interpretation, other studies have reported age-related decreases in impulsivity, sensation-seeking (Zuckerman et al., 1978; Willems et al., 2003), risky decision-making (Di Rosa et al., 2017), and reward sensitivity (Eppinger et al., 2012). As individuals become older, they may be less motivated by monetary reward and thus less vigorous in initiating reward-seeking actions. However, our findings do not imply a general age-related decline in motivation for rewards as there is evidence of enhanced sensitivity to social rewards in older, as compared to younger, adults (Rademacher et al., 2013).

Accuracy in rewarded no-go trials was found to improve with age, indicating opposite behavioral effects of age on inhibiting as compared to executing an action. Similar findings have been reported with other tasks. For instance, age was associated with increased inhibition as indexed by the reduced tendency to draw from the disadvantageous decks during the Iowa gambling task (Cauffman et al., 2010). Older adults also showed better avoidance learning in a probabilistic selection task (Frank and Kong, 2008), Digit–Symbol Substitution test, and Spot-a-Word test (Eppinger and Kray, 2011) compared to the younger counterparts. However, previous studies employing the GNG task in a neutral context (i.e., without reward) did not report age-related effects on inhibition of action (Schulz et al., 2007; Kubo-Kawai and Kawai, 2010). The current findings, therefore, may be specific to behavioral contexts with a reward contingency. Taken together, our work offers behavioral evidence for contrasting modulations of age on motivated action and inhibition of action.

### Age-Related Alterations in Neural Processes Underlying Rewarded Action

Consistent with the behavioral results, we found age-related attenuation in activity during rewarded action in regions involved in motivation and behavioral regulation, including the insula, MFG, rACC, and dACC. The rACC has been implicated in the flexible regulation of goal-directed behaviors (Kolling et al., 2016), particularly those involving reward-based cognitive control (Shenhav et al., 2013). The rACC responds to decision-making and problem-solving during reward-related contingencies (Hampton and O’Doherty, 2007; Amiez et al., 2012; Payzan-LeNestour et al., 2013). Using similar behavioral tasks, other imaging studies also found increased rACC responses to motor actions and action preparation (Watanabe et al., 2002; Schulz et al., 2011). Single-unit recordings of the human ACC further showed that ACC neuronal activity not only reflected changes in reward outcomes but also predicted motor movements during a sequential two-choice selection task (Williams et al., 2004), again in support of the role of the rACC in motivating actions.

Age also negatively modulated the activity of the anterior insula, MFG, and dACC. Work in both non-human primates and humans has implicated these regions in goal-directed behaviors. Executing an effortful action to obtain rewards in various behavioral tasks, including the GNG, has been associated with increases in the activity of the insula (Asahi et al., 2006; Dixon and Christoff, 2012), MFG (Bjork and Hommer, 2007; Rademacher et al., 2010), and dACC (Williams et al., 2004; Hayden and Platt, 2010). As these regions are also involved in reward processing, the decreased activations during rewarded action may reflect reduced reward sensitivity with age. As the rACC and dACC have been proposed to be involved in the reward response and decision-making during motor processing, respectively (Bush et al., 2002; Marsh et al., 2007), they likely interact to guide motivated behaviors (Rogers et al., 2004).

The diminished activity of the ACC, MFG, and insula may be related to the loss of structural integrity in these regions during aging. Indeed aging was found to significantly reduce the gray matter volume (Vaidya et al., 2007) and the metabolic activity, as measured by glucose uptake (Pardo et al., 2007) and blood flow (Meltzer et al., 2000), of the rACC. Aging is further associated with altered molecular profiles, as reflected by the loss of dopamine D1 (MacDonald et al., 2012), and D2/D3 (Kaasinen et al., 2000) receptors in the ACC. As dopamine plays a central role in the reward mechanisms (Schultz, 2007), reduced dopaminergic signaling may exert a negative effect on the initiation of reward-directed actions. Gray matter volume loss (Kalpouzos et al., 2009; Peelle et al., 2012) and metabolic reduction (Petit-Taboue et al., 1996) were similarly found in the MFG and the insula in older adults. The relationship between a decline in structural integrity, attenuated brain activity, and behavioral outcomes poses an interesting avenue for further research.

### Age-Related Alterations in Neural Processes Underlying Rewarded Inhibition of Action

Contrary to our hypothesis, we found age-related decreases in activation to rewarded inhibition of action in the medial SFG and the PoCG, both of which also exhibited age-related attenuation in activity during rewarded action. This finding indicates shared neural substrates between motivated action and inhibition of action in relation to aging. Accordingly the SFG has been implicated in the regulation of goal-directed behaviors and cognitive control (Konishi et al., 2003; Floden and Stuss, 2006; Hu et al., 2016), suggesting an important role in both action execution and inhibition. As part of the somatomotor cortex, the PoCG is involved in motor functioning in both humans (Raposo et al., 2009) and non-human primates (Iwamura and Tanaka, 1996). The age-related decrease in activation in this region may be associated with the slowing in motor processing as observed in the go performance in the current work.

Aging has been shown to negatively impact the structural integrity, including both the gray and the white matter, of the SFG (Raz et al., 1997; Oh et al., 2014) and PoCG (Raz et al., 1997; Minkova et al., 2017). Loss of gray matter may diminish their roles in action regulation, potentially leading to reduced activations to no-go responses as currently observed. This interpretation is in line with previous evidence of impaired cognitive control in older individuals (Braver and Barch, 2002; Andrés et al., 2008; Paxton et al., 2008). Nevertheless, it is important to note that we did not find a significant relationship between task performance during no-go trials and brain activity. Furthermore, the age effects on inhibition of action were most prominent for the nickel trials, yet the age-related diminution in activity during inhibition of action was observed for the dollar trials but not for the nickel trials. These findings suggest the complex influence of age on behavioral inhibition. Additionally, as no-go trials were less frequent than go trials, the biased incentivization may have rendered the no-go trials less salient than the go trials.

We found negative age effects in modulating the activities of shared regions, including the medial SFG and the right PoCG, during action and inhibition of action. Age was associated with poorer go performance but superior no-go performance. As such, the attenuation of these regional activity, particularly in the PoCG, may have opposite impacts on go and no-go trials. The PoCG has been associated with motor processing (Hyvärinen and Poranen, 1978; Woods et al., 2014). Thus, it is plausible that the lack of motor-related activation leads to a slower response initiation, making it less challenging to inhibit such response in older individuals. Indeed in regions associated with cognitive motor control, including the medial SFG (Rushworth et al., 2004; Sumner et al., 2007), diminishing activity with age may reflect less conflict between opposing actions (e.g., go vs. no-go). It is worth noting that the medial SFG has been shown to be functionally connected with motor regions (Zhang et al., 2012), putatively to modulate goal-directed movements. As the go response becomes weaker during aging, the amount of resources needed to inhibit a pre-potentiated action likely decreases, leading to the reduced need for motor control and recruitment of motor activities.

## Limitations and Conclusion

Findings from the current study should be examined with consideration of its limitations. Specifically, the high accuracy rate of the no-go trials indicates a relatively less challenging task condition. As the response window was titrated to the go response, no direct manipulation of the no-go trials was possible. Nevertheless, we examined the neural activations to action inhibition (i.e., contrast NGS dollar > NGS control) and found typical regions implicated in inhibitory control, including the MFG and the ACC (Supplementary Figure S2), indicating that the task was successful in eliciting the neural processes of inhibition. Additionally, given the insufficient number of no-go error trials in the nickel condition, we were unable to assess the brain substrates underlying reward loss sensitivity during inhibition in relation to age.

In sum, we found that brain activities underlying rewarded action and inhibition diminish with increasing age, potentially reflecting a broad attenuation in the neural processes involved in the integration of motivation and behavioral regulation. The regions previously implicated in cognitive control and reward processing, such as the MFG, ACC, and insula, all showed an age-related reduction in activity. The reduced brain activity mediated the effects of age on prolonged reaction time during rewarded vs. control go trials, suggesting more conservative responses in reward-seeking action beyond age-related motor slowing. These findings add to the neuroscience literature of motivated behaviors in healthy aging.

## Data Availability Statement

The datasets generated for this study are available on request to the corresponding author.

## Ethics Statement

The studies involving human participants were reviewed and approved by Yale Human Investigation Committee. The patients/participants provided their written informed consent to participate in this study.

## Author Contributions

C-SL, IL, and HC designed the study. TL collected and analyzed the data. All authors wrote the manuscript.

## Conflict of Interest

The authors declare that the research was conducted in the absence of any commercial or financial relationships that could be construed as a potential conflict of interest.
