# Supplementary material for: Age-Related Changes in the Neural Processes of Reward-Directed Action and Inhibition of Action
Source: Front Psychol. 2020 Jun 10;11:1121. doi: 10.3389/fpsyg.2020.01121 (PMC7298110; doi:10.3389/fpsyg.2020.01121)
Supplement: Supplementary file 1 [file Data_Sheet_1.PDF]

Le et al.'s Age-Related Changes in the Neural Processes of Reward-Directed Action and Inhibition of Action

Supplementary information

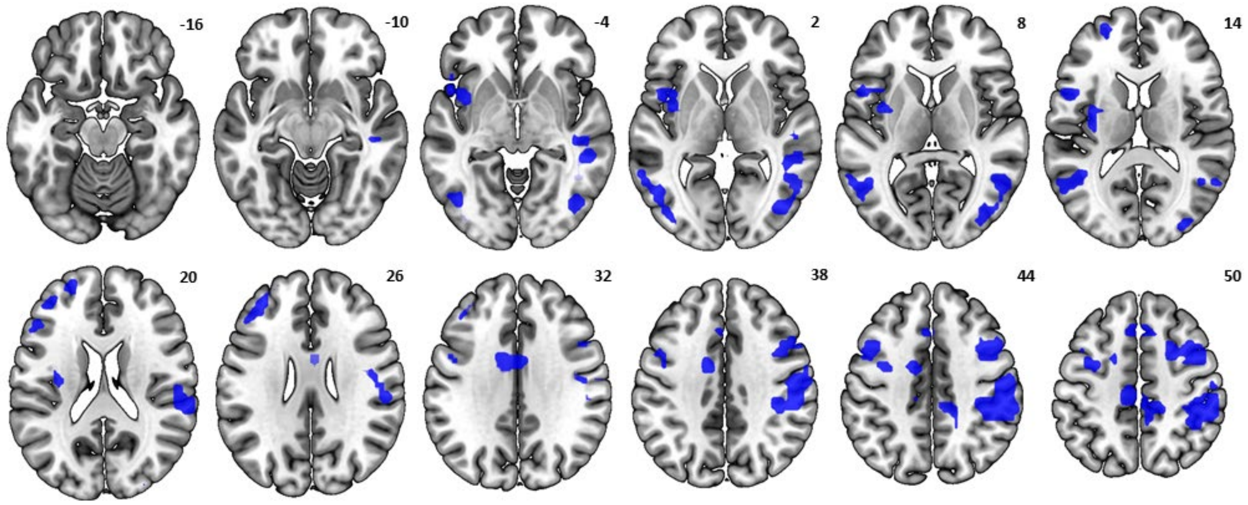

Response figure 1: Multiple regression of GS nickel > GS control against age showed significant negative activations in the left insula, dorsal anterior cingulate cortex, bilateral middle frontal gyrus, bilateral superior temporal sulcus, left postcentral gyrus, and bilateral occipital cortices.

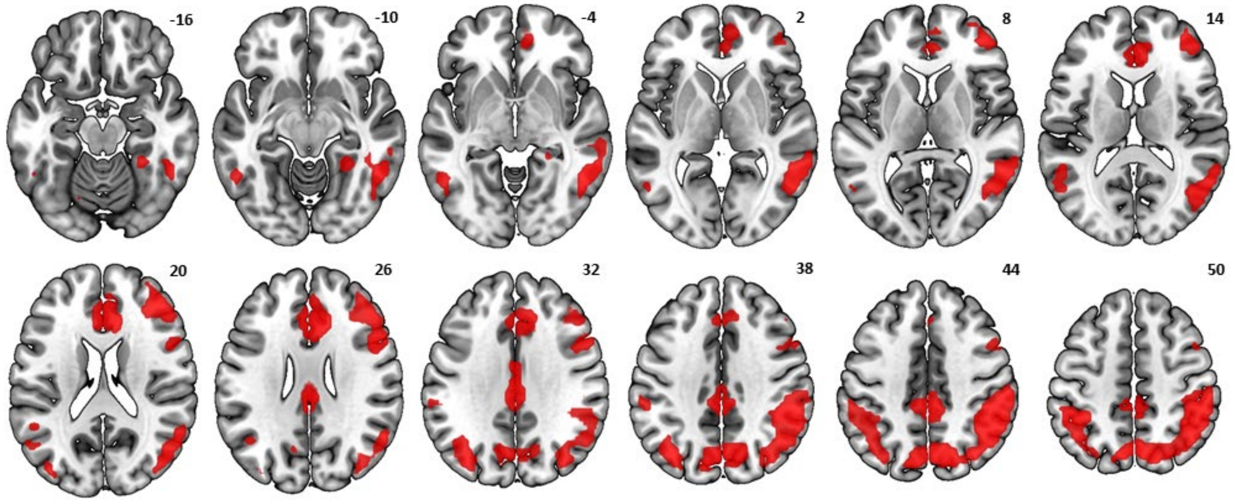

Figure S2: Brain activations to inhibitory control. The contrast NGS dollar > NGS control showed significant activations in the right middle frontal gyrus, dorsal anterior cingulate cortex, mid/posterior cingulate cortex, and a cluster containing bilateral postcentral gyrus and superior parietal lobule. Activations significant at voxel $p < .001$, uncorrected in combination with cluster $p = .05$ FWE.

Table S1: Mean (SD) of number of trials per block

	Block 1	Block 2	Block 3	Block 4
GS dollar	19.5 (6.2)	18.6 (5.6)	19.2 (7.4)	16.7 (10.3)
GE dollar	5 (4.9)	5.5 (5.3)	5 (5.8)	4.3 (5.2)
NGS dollar	10.9 (4.1)	11.5 (3.3)	10.2 (4.0)	8.4 (5.1)
NGE dollar	1.2 (1.3)	1.1 (1.4)	1.1 (1.5)	.8 (1.5)
GS nickel	15.2(6.6)	14.3 (6.9)	12.3 (7.0)	10.9 (8.9)
GE nickel	9.1 (5.4)	9.9 (5.7)	10.4 (6.6)	8.7 (7.9)
NGS nickel	10.6 (3.6)	11.8 (3.4)	11.4 (4.3)	8.6 (5.2)
NGE nickel	.8 (.9)	.5 (.7)	.6 (.9)	.5 (.9)
GS control	34.2 (9.4)	N/A	N/A	N/A
GE control	14.4 (9.0)	N/A	N/A	N/A
NGS control	21.7 (4.1)	N/A	N/A	N/A
NGE control	2.1 (2.2)	N/A	N/A	N/A
